# 3D Technology Development and Dental Education: What Topics Are Best Suited for 3D Learning Resources?

**DOI:** 10.3390/dj8030095

**Published:** 2020-09-01

**Authors:** Paulina Poblete, Sean McAleer, Andrew G Mason

**Affiliations:** 1 Escuela de Odontología, Facultad de Ciencias, Universidad Mayor, Chile; 2 Dundee Dental School, University of Dundee, Scotland DD1 4HR, UK; a.g.mason@dundee.ac.uk; 3 Centre for Medical Education, University of Dundee, Scotland DD2 4BF, UK; j.p.g.mcaleer@dundee.ac.uk

**Keywords:** three dimensional, educational technology, dental education, needs assessment

## Abstract

The aim of this study is to identify topics (knowledge and skills) from the dental curricula that would benefit from having a 3D learning resource using an exploratory sequential design method. The first phase targeted stakeholders from a Scottish dental school. Seven focus groups and three interviews disclosed 97 suitable topics for 3D technology development. These results were used to construct a survey that was sent to final year dental students, newly dental graduates and academics from three Scottish universities. The survey asked participants to rank each item based on the perceived benefit that a 3D learning resource would have for dental education. Results revealed that detailed anatomy of the temporomandibular joint, dental anaesthesiology, dental clinical skills techniques, dental occlusion and mandibular functioning were top priorities. Gender differences only appeared in relation to ‘Extraction techniques: movements and force’ (*p* < 0.05), this topic was considered to be more beneficial by females than by males. No statistical difference was found when comparing results of graduates with undergraduates. These results serve as a starting point when developing a new 3D technology tool for dental education, considering users demands and perceived needs has the potential to benefit dental students and dental education directly.

## 1. Introduction

The arrival of social media, mobile devices, personal computers, clinical technologies and visual technologies have modified how and where education occurs. These technological resources allow students to access information easily [1], to re-use learning materials [2], to study at a distance [3,4], and simulate training environments [5,6]. While limitations still exist, mainly because of the costs involved in the acquisition of this technology, dental education has now incorporated technology-based resources into its training and one key example is 3D technology [7]. Moreover, current curricular trends in dentistry consider simulation-based learning as an essential part of training [8]. Therefore, the development of technological resources is in high demand.

Several health science education publications describe the development of new resources using 3D technology [9,10,11,12,13,14,15,16,17,18]. Dentistry is not the exception and 3D technology has gained importance in the last 20 years. For example, a search in Scopus including the terms “3D” and “dental education” reveals only one paper in the year 2000 containing those terms; while 14 appeared in the year 2019 and 11 have been published so far in 2020. A review of the literature shows that 20 papers describe new software or novel uses of 3D technology for dental education [19,20,21,22,23,24,25,26,27,28,29,30,31,32,33,34] and 10 focus on students’ perception of the use of 3D tools [19,30,35,36,37,38,39,40]. However, only eight were comparative studies [35,38,40,41,42,43]. Recently, 3D printing has been successfully used for producing anatomic models which serve to simulate clinical scenarios for dental education [44,45].

When studying the use of these technological resources for education it is important to see the complete scope of the topic and how terminology is used. For example, the term ‘3D’, can refer to different types of technologies that depend on the context where it is used. A 3D model may or may not be an animation, and it can or cannot be part of a simulation system. A haptic device may have a virtual reality scenario that can be computer-based or attached to hardware that allows the simulation to occur. Considering this, it is necessary to look at the use of virtual reality in dentistry. It has been said that virtual reality is an innovative resource that in some areas of knowledge can bridge the gap between theory and practice [46]. The mixture of three key elements: 3D space, a visual representation of the user and the interactive chat produce the illusion of being immersed in a virtual world [47]. The use of virtual reality for dental education has been recently analysed revealing the existence of several virtual systems, however, the authors conclude there is still the need for further evidence surrounding their use and development [48].

Expert opinion suggests there should be a common approach to identifying students’ needs before developing a 3D digital [12,14,15,16,17,28,49,50]. However, few studies have addressed these needs before developing any new technological resource. Marsh et al. [51] surveyed 36 students at the University of Cincinnati Medical School about areas of learning that cause difficulty and the results revealed that embryology was one of the recurrent topics mentioned by the students. However, their sample was small and there were few details about the methodology and the construction of the survey. Usually, the development of 3D learning resources is justified by their reusability factor which in the long term makes them less expensive than traditional methods [52,53,54] and the fact that their use involves few ethical issues e.g., cadaveric dissections [55]. While these arguments are indeed valid, they do focus on providers’ needs rather than on the students’ needs.

This study aims to identify topics from the dental curricula (knowledge and skills) used by the University of Dundee that potentially would benefit from 3D virtual animations or simulations in the context of dental education.

## 2. Materials and Method

Two phases were planned using an exploratory sequential design described by Creswell [56]. The methodology selected helped to obtain a comprehensive view of the needs for 3D resources for dental education. The first phase was planned as focus group sessions and the second phase as a survey. The literature shows that data collected by means of focus groups are valuable for the construction of surveys [57,58,59]. Additionally, this methodology has been successfully used in medical education [60].

The first phase identified the knowledge components and skills that might benefit from a 3D format for dental teaching; the second phase prioritised the topics, by means of a survey, in order to produce a short list of topics suitable as 3D digital resources. Ethical approval was granted for this study by the University of Dundee Ethical Committee (UREC: 13084, 05/08/2013.).

### 2.1. Phase 1: Focus Groups and Interviews

The exploratory purpose of the focus groups [61] served to collect participants’ opinions towards what type of knowledge (facts, procedures, concepts and principles) and skills (cognitive and motor) might benefit from a 3D virtual format. Thirty-minute sessions were designed following recommendations encountered in the literature [62]. Five to six participants were invited per session in order to ensure sufficient data and a fluent conversation. The central question driving the sessions was: ‘What are the knowledge components and skills that would benefit from being taught using a 3D virtual format?’ Participants’ answers were transcribed by the moderator and the scribe as soon as answers arose, therefore recording was not considered necessary for data collection. At the end of the session, the listed items were reviewed by the whole group to ensure completeness of data. All data were transcribed to Microsoft Excel using a laptop. Repetitions were excluded and similar items were grouped. Descriptive analysis of data was conducted. In Table 1 the template of the focus group can be seen explaining the structure of the sessions.

The target population were dental students and academics from the University of Dundee. A sample of undergraduates from each academic year and lecturers from different areas of dental specialisation were used following a convenience sampling strategy. The study excluded first-year dental students as their experience was considered too limited at the scheduled time of the sessions. Participation was voluntary and every participant gave written consent after being given information about the study. In total, seven focus groups sessions were conducted; four with undergraduate students, one with postgraduate students and two with dental academics. On three occasions only one individual showed up to the scheduled focus group session; in those cases, the moderator conducted an interview following the same structure as the focus groups. In total, three individual interviews were carried out.

### 2.2. Phase 2: Survey

A survey was used to rank items obtained in Phase 1 as being best suited to develop 3D digital educational resources. The survey targeted 4th and 5th year undergraduate students, postgraduate students, dental graduates, and dental academics from three Scottish universities (Universities of Dundee, Glasgow and Aberdeen).

**Figure 1:**
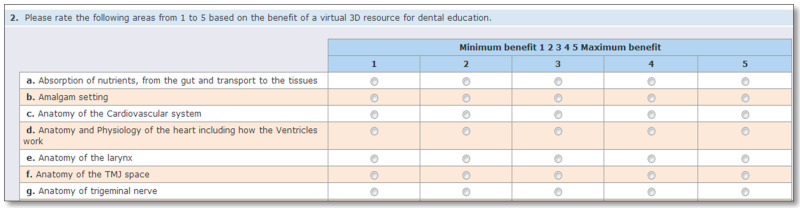
Extract of the survey.

The survey was constructed using OnlineSurveys, (former Bristol Online Survey BOS) and asked participants to rank each item based on their perceived benefit to have it as a virtual 3D format resource. A five-point Likert scale was used where 5 represented ‘Maximum Benefit’ and 1 ‘Minimum Benefit’. An extract of the survey can be seen in Figure 1.

Demographic details were also collected. The dissemination of the survey was coordinated by each University. A total of 767 emails were sent, each containing an invitation letter and an information sheet. The biggest concern was the potentially low response rate, so the survey was as brief as possible, and used an informal written format. Reminders and incentives were used as recommended in the literature [63]. Reminders were sent via email and two £50 amazon vouchers were offered as incentives.

For data analysis the five-point Likert scale responses were categorised into two groups: ‘beneficial’ (grouping scores 4 and 5) and ‘non-beneficial’ (grouping scores 1, 2, and 3). An item was considered ‘highly beneficial’ when ≥80% of the responses were rated 4 or 5. In total 17 items were considered ‘highly beneficial’ and statistically analysed. Fisher exact test was used to compare genders and graduates versus undergraduates. 

## 3. Results

**Figure 2:**
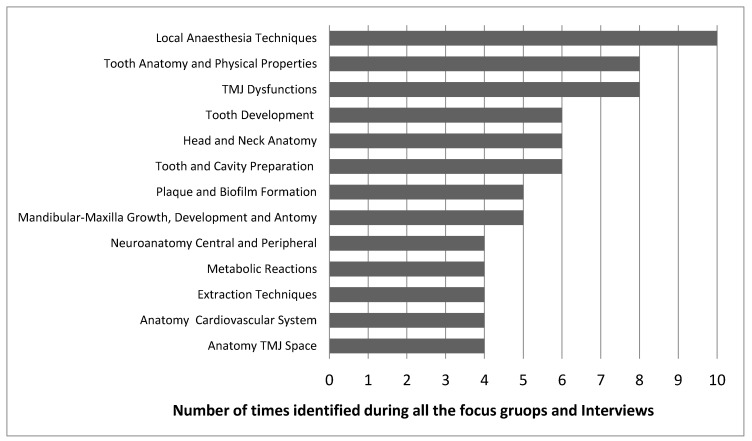
Most recurrent Items from all the focus group sessions and interviews.

In total, 36 volunteers participated in the first phase of this study, generating a list of 198 items. After excluding repetitions, a final list of 97 items was obtained. The 13 most recurrent items are shown in Figure 2.

One hundred and twenty-eight responded to the survey, a 17% response rate (84 females and 44 males). The 97 items were ranked based on their potential benefit. Table 2 shows both extremes of the ranking list; the highest ranked (≥70%) items and the lowest ranked (≤30%).

The results revealed that detailed anatomy of the temporomandibular joint, dental anaesthesiology, dental clinical skills techniques, dental occlusion and mandibular functioning were top priorities. When broken down by gender only one of the highly ranked items: ‘Extraction techniques: movements and force’, was perceived as being more beneficial by females than by males (*p* < 0.05). Comparison between graduates and undergraduates revealed no statistical differences.

## 4. Discussion

The results of the focus groups revealed a variety of items ranging from basic science concepts to clinical procedures very specific to dentistry. It was noticed that the clinical procedures were more frequently mentioned among the responses of participants of the focus groups. From the 97 items named, a few had been already addressed by developers [10,13,14,19,20,29,32,33,35,64,65] e.g., tooth morphology, root canal related software, 3D study models, surgical procedures for dental implants. Tooth anatomy was among the most recurrent themes in the focus group, despite the fact that five studies [19,20,29,35,64] have highlighted the availability of a 3D learning resource for that purpose. Perhaps, the current respondents were unaware of these resources or maybe the actual resources did not comply with their current needs in terms of quality or expectations. Another topic that featured high on the request list was head and neck anatomy, an area that has been addressed by several authors [9,13,14,65]. Head and neck anatomy 3D software package was developed by Anderson et al. [32,33], yet it still seems to be an area that need more exploration of its use.

Overall, the focus group results suggest that participants believed that less emphasis should be placed on topics related to basic science and more attention should be given to applied dental skills. It could be argued that these results might be influenced by the fact that the sampling focused on students from 2nd year onwards. As focus groups were conducted in September (beginning of academic year), first years were not invited as their familiarity with the career was too limited at that time. However, no concerns were perceived by the authors, as the final list contained a variety of topics representing the complete dental curricula which served to build a comprehensive survey.

Results of the survey revealed that ‘Anatomy of the Temporomandibular Joint’ was ranked the highest from those areas of the anatomy of the head and neck. Despite the existence of many of 3D technological tools for anatomy education [66], the results of the present study reveal a strong need for 3D learning resources specifically related to anatomical areas relevant to dentistry. One of the reasons might be that current models used in teaching lack sufficient detail to satisfy users’ needs [66].

Other highly ranked items were ‘root canal treatment’ and ‘cavity preparation’; both important clinical skills for newly qualified dentists. Dentistry is a hands-on profession; thus it is not surprising that many of the items mentioned in this study have a strong relationship with clinical procedures and skills. Similar observations were made by Murray et al. [67] in a study that asked new graduates about possible improvements for undergraduate curricula. To comply with the hands-on nature of dentistry, haptic systems have been developed mainly addressing cavity preparation [5].

‘Dental anaesthetic techniques’ was another highly ranked item. Anaesthetic techniques are complex to learn and require excellent clinical skills, in combination with sound anatomical knowledge. In many aspects its teaching is controversial, as in some schools the first local anaesthetic injection performed by a student is given to a classmate [68] or to a human cadaver [69]; both of these practices are not free of ethical issues. It has been reported that dental anaesthesiology education varies across many universities [68] because of its complexity. There are many factors that need to be controlled and having a 3D resource to help students visualise the anatomy and understand the technique before practicing might help build student confidence and competence.

Spatial awareness has been related to the capacity to manipulate 3D objects [70] and the literature is inconclusive when it comes to differences between males and females. Some authors believe there is no difference [71,72] while others support the idea that this ability is affected by gender [70] assigning better results to males than to females. The results of this study revealed statistical difference in only one of the highly rated items: extraction techniques, movements and force. Females considered that a 3D learning resource related with extractions techniques is much needed compared to the views of males as they did not rank that particular item as higher. These results are aligned with findings reported by Macluskey et al. [73], which showed that females felt less confident when preforming dental extractions. Interestingly, evidence suggests that female dentists are more inclined to refer exodontia cases [74], which could be due to a lack of confidence with the procedure. This might explain why females perceived a greater need for 3D learning resources for extraction techniques. Strength and size of the operator might have some implications on this difference observed in regards with dental extractions confidence, yet no evidence was found to support these assumptions.

Perceptions of graduates and undergraduates showed no statistical differences among highly ranked items. These results suggest agreement among different stakeholders, regardless their experience indicating that there is a need for 3D learning resources in several areas of dentistry.

Interestingly, all groups indicated that the top priority item was: ‘Anatomy of the Temporomandibular Joint’. The importance of the temporomandibular joint (TMJ) is crucial for dentistry as it is important for occlusion and the function of the masticatory system. It has been suggested that a proper understanding of the TMJ should be mandatory before investigating the temporomandibular joint disorder [75]. Many authors have identified the challenges of teaching and learning TMJ disorders [76,77,78] especially with the lack of consistency in basic terminology [77,79].

Interestingly, it was noticed that almost double the number of female participants took part in the study (84 females versus 44 males). This could be a consequence of the feminisation of dentistry as a profession that has occurred in recent years [80,81]. This tendency was also observed in recent work by Macluskey [73] across UK dental students which showed that more women are choosing dentistry as a career path [80,81,82]. However, the exact number of how many females and males were invited constitutes a limitation of this study. Therefore, further studies are required to confirm the increasing number of females taking dentistry as a career path. Another limitation of this study was the low response rate to the survey, despite using techniques to increase participation. However, it has been established that the size of a sample is more important than the response rate [83]. Overall, results of the 128 respondents who completed the questionnaire disclosed the need to generate new 3D learning resources addressing multiple areas of dentistry. Additionally, data were normally distributed, so despite the response rate, data were suitable for analysis. The construction cost of detailed 3D models is one of the disadvantages of these resources [2]. Brenton [9] suggested that they can be expensive and time-consuming to generate, as their successful development demands special equipment. When it comes to the construction of a haptic simulator, again cost has been reported as the main issue [84,85]. This is not just important for dental education but for all the industry of 3D technology developers as resources are limited. This study serves as a starting point when planning the development of a resource for dental education. The authors highlight the importance of considering users need and seeking their opinion towards which topics might benefit their educational process before developing a new resource. Moreover, if the resource is meant for academic purposes the recommendation would be to include students when making the decision.

Even though this study represents the view of a small proportion of the dental community, it reveals that the demands of graduates and undergraduates are aligned. Technology, especially 3D technology, has not yet entered dental education as deeply as it potentially could. This is relevant and needs to be acknowledge by regulatory bodies such as the General Dental Council (GDC) (dentistry regulation body in the United Kingdom). In the latest learning outcomes declaration ‘Preparing for practice’ of the GDC [86], emphasis is given to the use of technology, yet very little links to simulation or training using three dimensional technology are declared in the document. Results of this study demonstrate the existence of a demand that could potentially satisfy the need to benefit the dental education process. Even though future research is needed to set up a global need of this resources, this constitutes a starting point showing the lack of embracement of these technologies inside the classroom.

Future research implies de need to determine the educational impact of 3D learning resources, in order to validate the relevance of production these type of resources for dental education.

## 5. Conclusions

The final conclusions of this study are:

- There are key topics that would benefit from 3D digital learning resources: anatomy of temporomandibular joints, detail anatomy of head and neck, dental anaesthesiology, dental clinical skills techniques, dental occlusion and mandibular functioning.
- Perception of need and benefit of 3D learning resource does not vary by level of formation (undergraduate/graduate). Gender analysis only revealed difference around one topic: ‘Extraction techniques: movements and force’.

## Figures and Tables

**Table 1:** Focus group template.

Focus Group Template
Opening (5 min) ○ Introduction of the session and presentation of the moderator and the scribe ○ Brief explanation of the aims of the project ○ Grounded rules ○ Confirmation of voluntary participation and seeking written consent Body (20 min) ○ The question driving the session was: ‘What are the knowledge components and skills that would benefit from being taught using a 3D virtual format?’ ○ Conversation build-up surrounding the question and emerging topics ○ Paraphrasing the question if needed ○ Data collection: notes taken by the moderator and the scribe Closure (5 min) ○ Moderator revise all the answers with the group ensuring all answers were collected ○ Closure and thank to participants for taking part

**Table 2:** Ranking of the perceived need for a 3D learning resource for dental education.

Ranking	Item	Percentage of Participant that Ranked the Item as Beneficial
1st.	Anatomy of the TMJ space	98%
2nd.	Root canal treatment model representing what happens inside the canal and how to determine the working length	92%
3rd.	Local anaesthesia techniques including the needle position, the tissues and how the needle passes through or close to.	90%
4th.	Anatomy of trigeminal nerve	91%
5th.	Concepts in occlusion such as Bennett angle, Bennett movement, condylar guidance, anterior guide, excursive movements	89%
6th.	Tooth and cavity preparation for crowns, onlays, inlays, ¾ crowns, endodontic access	88%
7th.	Head and neck anatomy	86%
8th.	Suturing techniques	86%
9th.	TMJ dysfunction; including for example clicking temporomandibular joints	86%
10th.	Impacted tooth identification and extraction techniques	85%
11th.	Extraction techniques: movements and force to extract the tooth	84%
12th.	Third molar extractions	84%
13th.	Surgical procedures for implants	84%
14th.	Course of cranial nerves until the innervated tissues	83%
15th.	Occlusion functioning and types	83%
16th.	Masticatory muscles anatomy and physiology	82%
17th.	Caries removal including tactile feedback	80%
18th.	Flap design	79%
19th.	Mandibular and maxillary development, growth and anatomy	77%
20th.	Use of elevators	77%
21st.	Mandibular fracture	76%
22nd.	Tooth anatomy and tooth physical properties	77%
23rd.	Denture design—3D model to design cobalt-chrome dentures	76%
24th.	Normal movements of the jaw and pathological movement	76%
25th.	Removal of large lesions such as cysts	76%
26th.	Space infections of the head and neck	76%
27th.	Model showing most common errors and bad decision making for restorative dentistry (e.g., errors in prosthesis design, errors in crown preparation)	74%
28th.	Development of the dental arch	73%
29th.	Le Fort fractures	73%
30th.	Periradicular surgery	73%
31st.	Mastication process	70%
32nd.	Biomechanics in orthodontics (tooth movement)	70%
33rd.	Indirect vision practice model	70%
34th.	Model in 3D of oral cancer development and progress	70%
81st.	Pathogenesis of diseases	30%
82nd.	Pharmacology—models of how drugs work in the tissues	29%
83rd.	Ear anatomy model	28%
84th.	Respiratory system model, including process of ventilation, perfusion	24%
85th.	Kidney anatomy	23%
86th.	Cell mitosis and meiosis	22%
87th.	Drugs clearance methods	21%
88th.	Physiology of the GI tract	21%
89th.	Exchange of oxygen in the alveolus	20%
90th.	DNA double helix	20%
91st.	Hormonal cycles. From hormone production to their action	20%
92nd.	Metabolic reactions—Pathways of chemical reactions represented as interactive models	19%
93rd.	Absorption of nutrients, from the gut and transport to the tissues	19%
94th.	Renal physiology	19%
95th.	Protein synthesis	15%
96th.	Functions of mitochondria and Golgi complexes	14%
97th.	Molecular interaction of amino acids synthesis	10%
